# Telemedical support for prehospital Emergency Medical Service (TEMS trial): study protocol for a randomized controlled trial

**DOI:** 10.1186/s13063-017-1781-2

**Published:** 2017-01-26

**Authors:** Ana Stevanovic, Stefan Kurt Beckers, Michael Czaplik, Sebastian Bergrath, Mark Coburn, Jörg Christian Brokmann, Ralf-Dieter Hilgers, Rolf Rossaint, TEMS Collaboration Group, Marc Felzen, Frederik Hirsch, Jürgen Wolff, Nils Lapp, Lothar Albrecht, Christof Koerentz

**Affiliations:** 1 Department of Anesthesiology, University Hospital RWTH Aachen, Pauwelsstr. 30, 52074 Aachen, Germany; 2 Emergency Medical Service, Fire Department, Stolberger Str. 155, 52068 Aachen, Germany; 3 Emergency Department, University Hospital Aachen, Pauwelsstr. 30, 52074 Aachen, Germany; 4 Department of Medical Statistics, University Hospital RWTH Aachen, Pauwelsstr. 30, 52074 Aachen, Germany

**Keywords:** Prehospital, Emergency medical system, Telemedicine, Teleconsultation, Remote treatment, Tele-emergency physician

## Abstract

**Background:**

Increasing numbers of emergency calls, shortages of Emergency Medical Service (EMS), physicians, prolonged emergency response times and regionally different quality of treatment by EMS physicians require improvement of this system. Telemedical solutions have been shown to be beneficial in different emergency projects, focused on specific disease patterns. Our previous pilot studies have shown that the implementation of a holistic prehospital EMS teleconsultation system, between paramedics and experienced tele-EMS physicians, is safe and feasible in different emergency situations. We aim to extend the clinical indications for this teleconsultation system. We hypothesize that the use of a tele-EMS physician is noninferior regarding the occurrence of system-induced patient adverse events and superior regarding secondary outcome parameters, such as the quality of guideline-conforming treatment and documentation, when compared to conventional EMS-physician treatment.

**Methods/design:**

Three thousand and ten patients will be included in this single-center, open-label, randomized controlled, noninferiority trial with two parallel arms. According to the inclusion criteria, all emergency cases involving adult patients who require EMS-physician treatment, excluding life-threatening cases, will be randomly assigned by the EMS dispatching center into two groups. One thousand five hundred and five patients in the control group will be treated by a conventional EMS physician on scene, and 1505 patients in the intervention group will be treated by paramedics who are concurrently instructed by the tele-EMS physicians at the teleconsultation center. The primary outcome measure will include the rate of treatment-specific adverse events in relation to the kind of EMS physician used. The secondary outcome measures will record the specific treatment-associated quality indicators.

**Discussion:**

The evidence underlines the better quality of service using telemedicine networks between medical personnel and medical experts in prehospital emergency care, as well as in other medical areas. The worldwide unique EMS teleconsultation system in Aachen has been optimized and evaluated in pilot studies and subsequently integrated into routine use for a broad spectrum of indications. It has enabled prompt, safe and efficient patient treatment with optimized use of the “resource” EMS physician. There is, however, a lack of evidence as to whether the advantages of the teleconsultation system can be replicated in wider-ranging EMS-physician indications (excluding life-threatening emergency calls).

**Trial registration:**

ClinicalTrials.gov, identifier: NCT02617875. Registered on 24 November 2015.

**Electronic supplementary material:**

The online version of this article (doi:10.1186/s13063-017-1781-2) contains supplementary material, which is available to authorized users.

## Background

German and some other European Emergency Medical Services (EMSs) consist of a dual system with two paramedics (with a professional training of 2 years) and one EMS physician accompanied by a further paramedic. The emergency call is received by the EMS dispatch center and answered by a specially trained paramedic [1] who dispatches either solely an ambulance with paramedics or simultaneously, in life-threatening cases, also an EMS-physician vehicle. This is called the rendezvous system. If an ambulance only is dispatched, but the on-site patient situation requires an EMS physician (e.g., a more life-threatening case, or the administration of a special medication, such as opioid analgesia for severe pain management or a drug to reduce systemic arterial pressure in case of severe arterial hypertension, is required), then an EMS physician can be requested by the paramedics on site. The German nationwide network of ambulance locations shows a significantly higher density than that of emergency-physician locations [2]. This is due to the fact that national law and local regulations of the 16 states of Germany define the maximal time frame for EMS aid. Hence, the paramedics usually arrive on scene several minutes earlier than the EMS physician. The paramedics must start with the primary emergency medical care using their own initiative, but within the restrictions of the German law and local regulations. Perhaps unsurprisingly, the time delay until the arrival of the EMS physician is more pronounced in rural than in urban areas and can jeopardize patients care by the omission of essential physician-dependent treatments. Increasing numbers of emergency calls, vacant EMS-physician posts due to lack of available emergency physicians, and the increasing workload of the EMS physicians hinder the disposability of EMS-physician treatment [3]. Paramedical teams alone cope with 56% of the current emergency calls in Germany, the remaining 44% require an additional EMS physician [2]. This ratio depends on the region and the ratio shifts to requiring more EMS-physicians in rural areas in comparison to urban areas. Furthermore, in Germany the arrival time of the EMS physician to the emergency scene has increased considerably in 95% of emergency cases during the last 20 years [4–6]. This time interval increased from 18.9 to 28.2 min between 1995 and 2012 and was more than 20 min in 17% of the cases in 2012. The obligatory EMS response time in the German state of North Rhine-Westphalia of 8 min for the first EMS unit in urban areas, and the 12-min response time in rural areas is based on the recommendations according to the law (RettG NRW). However, the data reveal that the time interval between the arrival of the EMS ambulance and the EMS physician is frequently prolonged. In addition, as recently shown in the German state of Rhineland-Palatinate, some EMS-physician sites do not provide continuous operational readiness due to the aforementioned shortage of physicians [7]. Although there is a lack of systematic data for all German states, it is obvious that similar conditions are widespread. In these cases EMS physicians from more distant locations, or helicopter emergency medical services, mostly during the daytime, are required. This circumstance further prolongs the response times of EMS physicians and treatments are restricted to those administered by paramedics until their arrival. In many cases the paramedics are not able, or not allowed, to administer adequate aid (i.e., administration of opioids or performing invasive procedures) [8].

In summary, Germany has a well-established ground-based EMS, but five problems remain:

- Prolonged emergency response times with delayed treatments by EMS physicians
- Increasing numbers of emergency calls
- Shortages of EMS physicians
- Outdated communication systems
- Regionally different but improvable emergency treatment quality

### Mobile prehospital EMS teleconsultation

Telemedicine networks between medical personnel and medical experts have been shown to be beneficial for the quality of service that they supply in many medical fields. National and international teleconsultation is increasingly used for emergency care of stroke patients between hospitals with and without specialized stroke units [9–13]. It has also been shown that the real-time use of digital observation cameras is more favorable than using telephone consultation [12, 13]. Furthermore, transmission of a prehospital 12-lead electrocardiogram (ECG) and telephone consultation with a cardiologist improved the emergency treatment and the outcomes of acute myocardial infarction patients [14–18]. Other prehospital teleconsultation systems in emergency medicine are rare and have only been used in pilot projects [19, 20]. The American Heart Association (AHA) emphasizes the use and scientific evaluation of teleconsultation systems in prehospital emergency care [21–23]. The German research project “Stroke Angel” showed that the use of a tablet computer, with structured collection of stroke-specific patient data and its automatic forwarding to the hospital, decreased the prehospital process time of acute stroke management by half [20]. However, one disadvantage of this concept is the very specific focus on one medical condition.

The obvious need for a safe and widely usable tele-emergency system encouraged us to develop a holistic, multifunctional, algorithm-based, mobile teleconsultation system as a complementary structural element to the ground- and air-based EMS.

### Routine use of the EMS teleconsultation system in Aachen

After the favorable experience of the two pilot studies [19, 24], the EMS teleconsultation system was introduced step-wise into routine use, complementary to the ground- and helicopter-based EMS in Aachen. Since 2014 the costs have been covered by health insurance funds. All ambulances have been equipped with the teleconsultation system since 2015. To date, the teleconsultation EMS physician (tele-EMS physician) has been mainly used for specific cases, such as hypertensive emergency, stroke, and dislocated fractures, to which solely paramedics have originally been dispatched. The decision to involve a tele-EMS physician is made by the paramedics on scene based on standard operating procedures (SOP) devised by the EMS medical director, after obtaining verbal informed consent from the patient. Additionally, the paramedics decide on their own whether they require a physically present EMS physician on scene. Therefore, the tele-EMS physician is used either during the time gap until the arrival of the physically present EMS physician or the tele-EMS physician provides medical and organizational advice without a physically present EMS physician. This offers the opportunity for the paramedics to administer medications and perform invasive procedures under supervision by the tele-EMS physician, which is otherwise not possible under national laws and local regulations. For example, paramedics are not allowed to administer opioids without a delegation by a physician. If adverse events (AEs) occur, the tele-EMS physician can give advice to prevent further possible patient harm. Thus, the teleconsultation system in Aachen protects patients from delayed physician-dependent treatments and provides a high-quality medical therapy by using software-based, guideline-conforming treatment. Delegation of opioid administration by physicians to nursing stuff is usual in hospital, but unusual in EMS. Severe pain or other life-threatening conditions require prompt and accurate medical treatment. In one pilot study, conducted in the EMS of the central state of Hessen, an EMS physician delegated by phone the administration of morphine by paramedics in 172 patients with limb trauma; this resulted in no life-threatening AEs and their pain was significantly reduced [25]. In contrast to the in-hospital delegation by phone and in the latter study, the tele-EMS physician in Aachen monitors (ECG, pulse oximetry, blood pressure measurement, voice communication with the paramedics) the patient continuously during the treatment until the patient is handed over to another physician (EMS physician or physician in hospital). This system holds the potential to improve patient safety by giving the paramedics online medical control. Concurrently, the obligatory indications for dispatching of conventional EMS physicians have been restricted since March 2015, as a tele-EMS physician is available for secondary medical and organizational advice at any time. This refers, for example, to the emergency cases where a physician-decision regarding drug administration is required. Meanwhile, 4219 patients (1 April 2014 to 31 March 2016, worldwide the largest case series), were routinely treated using this EMS teleconsultation system in Aachen. Until now, the primary dispatching of the tele-EMS physician directly by the dispatching center has been waived. However, analysis of the severity levels of cases to which the EMS paramedics responded with the secondary use of the tele-EMS physician shows similar severity levels as the primarily dispatched EMS physician-staffed responses. Furthermore, the use of the tele-EMS physician instead of a conventional EMS physician did not show any complications and the quality of the medical history survey, the medical treatment and the documentation of “Tracer” diagnoses, such as stroke and myocardial infarction, improved. Additionally, the engagement time of the physician decreased by half with the use of tele-EMS. Our experience from the routine use of the teleconsultation EMS system in Aachen – excluding critical emergency cases such as severe trauma, cardiopulmonary resuscitation and advanced airway management – shows that this system may provide an equal or even better quality of care than the conventional EMS system. Furthermore, this system enables more economical utilization of EMS-physician resources. A nationwide implementation of this teleconsultation EMS system in all dual EMS systems requires proof that it is not harmful and shows some advantages in cases where primarily a tele-EMS physician is required (excluding life-threatening emergency calls).

### Objectives and study design

**Fig. 1:**
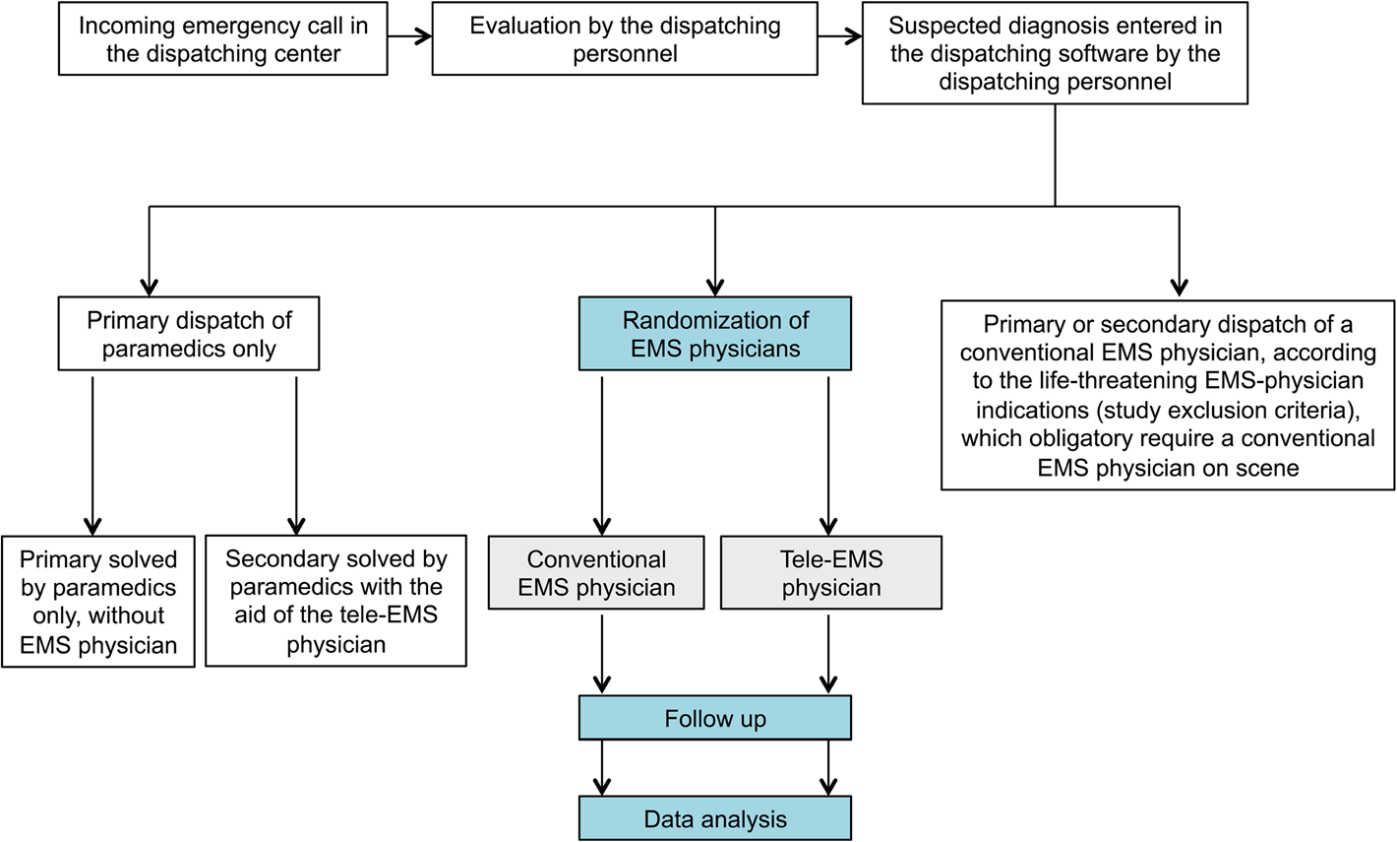
Study design. Flow chart of the study conduction in the clinical routine

The TEMS trial is designed as a single-center, prospective, randomized, interventional, open-label, two-arm, parallel-group, sequential noninferiority trial (Fig. 1). The purpose of this trial will be to assess:

- The quality of prehospital emergency care using the multifunctional teleconsultation system between paramedics and experienced tele-EMS physicians

### Specific primary objective

To determine if the primary usage of a tele-EMS physician compared to a conventional EMS physician on scene, in non-life-threatening cases, is noninferior with regard to the occurrence of system-induced patient AEs.

### Specific secondary objective

To determine if the primary usage of a tele-EMS physician compared to a conventional EMS physician on scene, in non-life-threatening cases, is superior regarding the quality of the medical history survey, medical treatment and documentation.

### Other secondary objectives

- To determine the different durations between the emergency call, the emergency response by a physician and the arrival time in the hospital
- To determine National Advisory Committee for Aeronautics (NACA) score classifications of the treated patients
- To determine the number of emergency cases which were handed over to the tele-EMS physician
- To determine the number of additional requirements of EMS physicians on scene in the case of primarily dispatched tele-EMS physicians
- To determine technical performance
- To determine the direct costs involved in the two systems
- To determine the acceptance of the teleconsultation system by the paramedics, the EMS physicians, the patients and the emergency room personnel
- Parallel to the randomized main study, we aim to retrospectively analyze all EMS cases which were excluded according to the exclusion criteria and treated by a conventional EMS physician during the study period
- To determine the frequency of tele-EMS-physician consultations by a conventional EMS physician
- To analyze additional data, which will be collected during a prepended “run-in” phase of 1.5–2 months

## Methods/design

### Study setting and design

This prospective single-center, randomized interventional, open-label, two-arm, parallel-group sequential trial will be conducted in the urban EMS in Aachen, Germany. This study protocol is reported according to the SPIRIT Statement and the SPIRIT Checklist is provided in Additional file 1.

### Eligibility criteria

#### Patient inclusion criteria

All non-life-threatening emergency calls, which do not obligatory require an EMS physician on scene and which do not solely require an ambulance staffed by paramedics, will be included in this study. This results in the following case for randomization:

- An emergency call is received by the EMS dispatching center in Aachen. The EMS dispatching personnel decide, after a structured interview with the caller, that there is not a life-threatening situation (according to the exclusion criteria), but a physician is required in addition to the paramedics

#### Patient exclusion criteria

All life-threatening emergency cases, where a physically present EMS physician on scene is obligatorily required according to German regulations. These include the patient’s condition and emergency case-related indications:

1. Patient condition-related indications:
  Apnea
  Acute respiratory failure
  Cardiocirculatory arrest
  ST-elevation myocardial infarction (STEMI)
  Unconsciousness
  Persistent seizure
  Life-threatening arrhythmia
  Major trauma
  Complex psychiatric disorder
  Age below 18 years
2. Emergency case-related indications:
  Major vehicle accident
  (Traffic) accident involving children
  Fall from a height (>3 m)
  Gunshot, knife, or blunt injuries to the head, neck or torso area
  Fires with reference to personal injury
  Explosion, thermic or chemical accidents with reference to personal severe injury
  High-voltage electrical accident
  Water-connected accidents (drowning, diving accident, fall through ice)
  Accidents involving hazardous goods
  Hostage-taking, rampage or other crimes with potential danger for human life (preventive deployment, police consultation)
  Immediate threat of suicide
  Pregnancy where delivery is imminent or expected very soon,

### Requirement of telemedical facilities

All routinely used EMS ambulances in Aachen are equipped and connected to a specific teleconsultation center. This system consists of four sections: the teleconsultation center, a server infrastructure, a fix-mounted in-vehicle PC and a portable autonomous communication device called the “PeeqBOX,” as previously described [26, 27].

### Teleconsultation center

The teleconsultation center is staffed by experienced EMS physicians from the Department of Anesthesiology of the University Hospital of RWTH, Aachen, Germany and physicians of the P3 Telehealthcare GmbH, Aachen, Germany. During the clinical routine one round-the-clock tele-EMS physician is required. In the course of this study an additional tele-EMS physician will be engaged. Minimal educational requirement for tele-EMS physicians comprises a minimal experience of 4 years in anesthesiology and intensive care medicine and an EMS-physician certification. Furthermore, additional qualifications such as European Resuscitation Council Provider and Pre-Hospital Trauma Life Support are required. All tele-EMS physicians must pass a specific training regarding the telemedical characteristics including communication principles. The teleconsultation center is located next to the dispatching center in the region of Aachen. Live vital data (curves and numerical data of ECG rhythms and, if needed, 12-lead ECG, pulse oximetry, end-tidal CO_2_ concentrations, blood pressure measurements) are transmitted to the tele-EMS physician. Solely the data transmission systems manufactured by GS Stemple (Kaufering, Germany), which are approved according to the medical devices law, are used. In addition to this diagnostic data, still-picture transmission and video transmission data are available to the tele-EMS physician. These data are explicitly used in addition to verbal communication with the paramedics and not for diagnostic purposes. The content of this visual data must be verbally verified. SOP, guidelines and drug databases are IT-based and provided to the tele-EMS physician. Standardized, context-sensitive and checklist-based documentation software is used in the teleconsultation center for all emergency cases conducted with the aid of a tele-EMS physician. Data transfer from the emergency scene, or the ambulance to the teleconsultation center, respectively, as well as the bidirectional audio connections are encrypted according to the state of the art. This enables the highest degree of interception protection during data transmission. To further improve data safety, all data are marked with an electronic key. This key verifies the authenticity of incoming data in the teleconsultation center and only accurate data become presented. Data storage occurs on specially secured servers with access restricted only to medical project management. Video data are not stored, but only streamed.

### General interventions for all patients

The patients will be randomized into two groups as shown in Fig. 1:

1. Conventional EMS
  A physically present conventional EMS physician on scene will treat the patients according to the memorized or carried SOP
2. Teleconsultation EMS
  The patients will be treated by the paramedics who are concurrently instructed by the tele-EMS physicians of the teleconsultation center according to the IT-based SOP, guidelines and drug databases

### Interventions—modifications

Premature discontinuation of the randomized treatment of a patient is only possible by the teleconsultation EMS group in three situations:

1. If a relevant technical defect occurs, the teleconsultation will be discontinued. Attempting to repair the teleconsultation system should be omitted during patient treatment. A conventional EMS physician will be dispatched immediately. A technical repair and diagnosis can follow after termination of the emergency case
2. If a patient waives their original informed consent after the beginning of the teleconsultation treatment, a conventional EMS physician will be dispatched immediately
3. If a situation develops within the treatment period, which requires a conventional physician according the exclusion criteria, a conventional EMS physician will be dispatched immediately

In the event of modifications or discontinuations of the study treatment, study participants will be retained in the study to enable data collection and preclude missing data.

### Outcomes

The participant timeline is shown in Additional file 2.

### Primary outcome measures

The primary outcome measure will include the rate of AEs depending on the specific treatment by an EMS physician or a tele-EMS physician. A Clinical Endpoint Committee (CEC) will perform the endpoint adjudication by blinded evaluation and assignment of AEs into intervention-related AEs, according to predefined criteria.

These intervention-related AEs are defined as follows:

- Immediate allergic reaction to drug administration due to incorrect survey of patients’ medical history (omission of the question for drug allergies)
- Intervention-related and immediate treatment-requiring hypotensive episode on scene (e.g., after wrong drug dosing or drug selection)
- Immediate intervention-related apnea or respiratory insufficiency on scene (e.g., after wrong drug dosing or drug selection)
- Intervention-related circulatory arrest within 24 h of EMS treatment (e.g., after wrong drug dosing, drug selection, or wrong referral to a hospital, which is not-specialized for the respective emergency case)

### Secondary outcome measures

The secondary outcome measures will record the specific treatment-associated quality indicators depending on the use of an EMS physician or a tele-EMS physician. These specific treatment-associated quality indicators are defined as:

- Quality of medical history survey (adherence to the guidelines)
- Treatment quality (adherence to the guidelines)
- Quality of documentation
- Duration of the physician engagement-time
- Fulfillment of predefined quality indicators for “Tracer” diagnoses:
  - Trauma
  - Stroke
  - Acute coronary syndrome
  - Pain control
  - Bronchial asthma
  - Chronic obstructive pulmonary disease (COPD)
  - Seizure
  - Sepsis
  - Hypoglycemia

- Correct prehospital diagnosis (comparison to the hospital discharge diagnosis)
- AEs (independent of the kind of EMS care, e.g., allergic reaction despite adequate survey of medical history, nonintervention related hypotensive episode, apnea or cardiac arrest)
- Premature termination of the telemedical or conventional EMS operation due to it being unnecessary
- Required conversion from a primarily dispatched tele-EMS physician to a conventional EMS physician
- Assessment as to whether a conventional EMS-physician operation could have been handled by a tele-EMS physician

### Other outcome measures

The following variables will be assessed in each randomization group:

- Time point of the first contact with a physician, time span between the emergency call and hospital arrival
- Seven-step National Advisory Committee for Aeronautics (NACA) severity score
- Proportion of conventional emergency cases which were passed to a tele-EMS physician (differentiated into medical need and lack of capacity)
- Assessment as to whether an EMS physician was even necessary for each emergency case
- Technical performance
- Survey of the paramedics, the patients, the EMS physicians and the emergency room personnel regarding their satisfaction with the EMS system used
- Death within 24 h and until day 30 of hospitalization, respectively, until discharge from hospital
- Death within 30 and 90 days after EMS treatment
- Discharge destination from hospital
- Intensive Care Unit (ICU) and hospital length of stay
- Frequency of tele-EMS contacting by a conventional EMS physician for any kind of advice
- Assessment of the medical education/experience of the involved physicians in each group
- Additionally, all outcomes will be assessed for patients who recruited during the run-in phase
- Retrospective analysis of the same data (excluding the prospectively collected satisfaction surveys and 30- and 90-day follow-up), as in the main study for the excluded conventional EMS physician cases. These data will be collected from the conventional EMS-physician protocols and the hospital database

### Participant timeline

A time schedule according to the SPIRIT figure is shown in Additional file 2.

### Run-in phase

One to two months prior to the main study, we will assess the same outcomes as in the main study.

#### Phase A: enrollment

The dispatching personnel in the EMS dispatching center in Aachen will screen all emergency calls for eligibility and enter a suspected diagnosis into the dispatching software.

#### Phase B: allocation

All non-life-threatening emergency calls, which do not obligatorily require an EMS physician on scene but cannot solely be resolved by the paramedics, will be randomized into the two intervention groups (conventional EMS physician and tele-EMS physician, respectively) automatically by the dispatching software.

#### Phase C: during EMS intervention, post allocation

Patients will be treated by both kinds of physicians according to the SOP and all operation-related data will be documented in a standardized EMS Documentation Form according to the recommendation of the German Interdisciplinary Society for Intensive and Emergency Medicine (DIVI). As usual in the present routine, the patients in the tele-EMS-physician group will be verbally informed about the use of a tele-EMS physician and the teleconsultation system. Written informed consent for study participation will be obtained as soon as possible during this phase until discharge from hospital.

#### Phase D: early follow-up, post allocation

A survey of the patients regarding their satisfaction with the EMS system used will be conducted after hospital arrival until discharge. Assessment of the outcome death within 24 h and until hospital discharge, respectively. Assessment of ICU and hospital length of stay and the discharge diagnosis of the hospital.

#### Phase E: late follow-up, close-out maximum until day 90

Additionally, we will assess mortality within 30 and 90 days, respectively.

#### Phase F: additional analysis, parallel to the main study

Retrospective analysis will be made of the nonrandomized, with conventional EMS-physician-treated patients. A satisfaction survey of the paramedics, EMS physicians and emergency room personnel will also be undertaken.

### Sample size

The sample size calculation is based on an assumed AE rate of 2% for the conventional physician-based EMS. The rate of 2% is based on our own analysis of 100 EMS-physician cases, as we could not find any information about EMS-related AEs in the medical literature. We assumed a noninferiority margin of 1.5% and allocated the overall 5% significance level to *K* = 3 (power (1 – *β*) 80%). Interim analysis will be performed according to the O’Brien and Flemming procedure [28]. The critical values, power and sample sizes for the group sequential design are given in Table 1.

**Table 1:** Statistical calculation based on the Farrington und Manning formula [40]

Information rate	Bounds accept H0	Bounds reject H0	Significance level one-sided	*α* spent	*β* spent	Power achieved	Stage *n1*	Sizes *n2*
0.333	-	3.471	0.0003	0.0003	-	0.0329	501.4	501.4
0.667	-	2.454	0.0071	0.0072	-	0.4424	501.4	501.4
1.0	2.004	2.004	0.0225	0.0250	-	0.8000	501.4	501.4

*H0* null hypothesis, *n* number

Using an allocation ratio of (*n2*/*n1*) = 1, the necessary sample size is 1504.2 + 1504.2 = 3008.4, thus resulting in a total sample size of 3010 patients. A fixed sample-size design would need *n1* = 1478.5 and *n2* = 1478.5. The expected total sample size under the alternative is 2531.7. A stop for futility is not planned.

### Recruitment

Beside our main study, we will initially perform a 1.5–2-month run-in phase to optimize the study conduction processes. During this period, we aim to enroll, randomize and follow-up around 300–600 additional patients who will be analyzed separately from the main study. This will enable process optimization and increase the compliance of the entire involved personnel. Thereafter, we will recruit 3010 patients, according to the sample size calculation of the main study. Patients will be recruited by the dispatching center in Aachen. This implies the evaluation of emergency call severity and exclusion of the life-threatening cases listed in a written procedure instruction. The impact of selection and allocation sequence bias will be determined with type 1 error.

### Randomization

The responsible biostatistician will select the most suitable randomization procedure to minimize the impact of selection and chronological bias on the test size based on a clinical evaluation scenario. Details of the randomization procedure will be described in a randomization report which will be kept concealed until closure of the database. The randomization sequence will be implemented into the dispatching software and will, therefore, remain concealed until all important data, including the suspected diagnosis of the next emergency case, are entered into this system by the dispatching personnel. Subsequently, the dispatching software will automatically randomize all patients with eligible suspected diagnoses into the two intervention groups and then the dispatching center personnel will assign the patients according to this proposal.

### Blinding

Blinding of the dispatching personnel, the paramedics on scene, patients and the physicians is not possible for practical reasons. Furthermore, the electronic Case Report Form (e-CRF) entering personnel cannot be blinded. The outcome assessors of the late follow-up variables and the CEC for the primary endpoint adjudication will be blinded to the kind of EMS intervention. As usual the Data Safety Monitoring Committee (DSMC) will not be blinded due to safety reasons.

### Unblinding procedures

There are no events expected to require unblinding of the outcome assessors.

### Data collection methods

The entire EMS personnel (including the dispatching personnel, the physicians and the paramedics on scene) will be informed about the study procedures and supervised by the principal investigator. The principal investigator will ensure local training of the entire EMS personnel to enhance the data collection quality and reduce bias. Furthermore, the assisting study personnel must be adequately qualified and informed about the study protocol, any amendments, and study-related responsibilities and functions. A study staff authorization log will be maintained. Every reasonable effort will be made to follow each enrolled and randomized patient until completion of study phase E.

### Participant withdrawal and loss to follow-up

Participants may withdraw from the study for any reason at any time. The follow-up period in this study is maximum 90 days. We do not expect many withdrawals or losses to follow-up until phase E. For phase E, we expect higher loss to follow-up data for the outcome variable mortality on day 90. The reasons for missing data will be recorded.

### Data management

SOP for data management will be implemented to ensure accurate, consistent, complete and reliable data. All collected data from a subject during the course of this trial must be filled and/or entered in the respective patient study Case Report Form (CRF).

As source data will serve:

- The standardized routinely created EMS files on scene (during both the conventional physician as well as the tele-EMS-physician operation)
- The additional EMS documentation files created in the teleconsultation center (including the transmitted patient vital data, e.g., ECG and blood pressure measurements)
- The routinely conducted EMS operation time recording in the dispatching center
- Data from the admission hospital database: diagnoses according to the *10th version of the International Classification of Diseases* (ICD 10), time points of the relevant diagnostic or therapeutic procedures (e.g., computer tomography, cardiac catheterization), laboratory data and severity scores of diseases (e.g., National Institutes of Health Stroke Scale for stroke patients)
- The clinical follow-up data, such as death after 24 h, or 30 and 90 days, respectively; the ICU and hospital length of stay and the hospital discharge diagnosis which are already collected for quality assurance reasons, based on the Law on Rescue Services of the state of North Rhine-Westphalia (RettG NRW, version of 18 March 2015); and the hospital discharge destination, will be collected from the hospital database

A high validity of the collected data can be assumed, as they are collected during the clinical routine and not specifically for the study. Furthermore, there will not be an additional burden for the medical personnel and any acquisition and storage of sensitive and confidential patient data, as all data will be entered pseudonymized into the CRF. According to a SOP, all relevant source data will be manually entered by the study personnel in a validated e-CRF. The respective patients’ paper-based CRF will contain the written informed consent with the date of subject information, a unique study and subject number.

Paper printouts of study data from an electronic database must be signed and dated by a member of the site staff to confirm the accuracy and completeness of data in the paper printout. Additionally, the monitor will sign and date the verified paper printout. The paper printout will be stored in the CRF. Retrospectively entered source data information on these paper printouts must be initialed and dated. Furthermore, any corrections of the original data require the drawing of a single line through the error and the dated signature and initials of the signatory. Hereby, the deleted entry should remain legible. The investigator will not falsify the data.

### Clinical Endpoint Committee

Blinded CEC members will perform the primary endpoint adjudication, to enhance the validity of the assignment of the four prespecified AEs into intervention-related or not. The CEC will consist of three members who are fully independent from the investigators and the sponsor.

### Statistical methods

To prove the noninferiority hypothesis, that the system-induced patient AE rate for treatment by the tele-EMS physician compared to the conventional EMS physician is not inferior by 1.5% of the (1 − *α*)% confidence interval for rate differences, will be calculated and accounted for in the interim analysis (Table 1). The hybrid analysis population developed by Sanchez will be used as the analysis population [29]. Further details will be given in the trial Statistical Analysis Plan before database lock. The secondary dichotomous parameters will be tested using the chi-square test and the continuous parameters by the *t* test assuming heterogeneous variances. A subgroup analysis will be performed according to the level of medical education/experience of the involved physicians in each group.

### Data monitoring

This study will be conducted in accordance with the approved protocol version, the ICH-GCP (International Conference on Harmonization of Technical Requirements for Registration of Pharmaceuticals for Human Use-Good Clinical Practice) principles, the Declaration of Helsinki, regulatory authority requirements and the SOP.

Qualified monitors from the sponsor Clinical Trial Center Aachen (CTC-A) will perform the monitoring visits according to the ICH-GCP principles and their SOP. Before the initiation of this study there will be one or more monitoring visits to check and clarify the prerequisites. The investigator is obliged to enable direct access to the source data and CRFs for study-specific monitoring, auditing and inspection by the competent Ethics Committee. The investigator will support the respective person and be available for questions. Regular monitoring visits to scrutinize random samples of data should discover problems and address discrepancies and verify that the data collection and documentation process is conducted in accordance with the ICH-GCP principles, the study protocol and the regulatory authority requirements. The following processes and data will be reviewed in a random sample of data during the monitoring visits:

1. Entries in the e-CRF
2. Adherence to the study protocol, the ICH-GCP principles, the Declaration of Helsinki, and the regulatory authority requirements
3. Integrity of the source data and the e-CRF entries
4. Accuracy of the documentation and report of AEs and serious adverse events (SAEs) within the required time periods
5. Patient identification, screening and enrollment log, study staff log and the monitoring log
6. Accuracy and completeness of the Trial Master File

Further monitoring details will be set forth in the monitoring manual.

### Data Safety Monitoring Committee and interim analyses

A formal DSMC will consist of three members with no competing interests who are fully independent from the sponsor and investigators. Comprehensive analyses of the documented AEs will be performed on a monthly basis. AEs will be assessed on specific forms. The frequency of the predefined AEs (please see “Primary outcome measures”) as well as nonintervention-related AEs (please see “Secondary outcome measures”) and resuscitation within 24 h of hospital admission will be referred to the DSMC. This committee will decide whether the AEs are intervention-related or not. If there appears to be a difference of 5% intervention-related AEs in one study-arm, then the DSMC must recommend premature study termination. Due to the complexity of the data, a useful interim analysis is planned after the inclusion of 1000 and 2000 patients, regarding the primary outcome variable. But if the analysis of the AEs indicates the need for an interim analysis, this will be conducted immediately.

### Harms

We expect an increase in patient safety with the support of a tele-EMS physician in emergency cases. Intensive standardized training of the entire EMS personnel regarding the teleconsultation system had already been performed before its implementation for routine use. This training focused on the transmission technology and on uniform and purposeful communication. This should enable a structured, efficient and safe transmission of the conversation content. Furthermore, legal matters, such as the informed consent of patients, the legal relationship between the paramedics and the tele-EMS physicians and liability risks, were discussed and clarified. A further safety measure is the support of the tele-EMS physicians by the software-based checklists. The advantages of checklist-based operations have been shown for patient safety within the World Health Organization (WHO) project: “Safe Surgery Saves Lives” wherein improved patient outcome was shown for elective as well as for emergency surgery with the checklist-based working [30–32]. The SOP for the teleconsultation system defines such checklists for the tele-EMS physicians, to ensure a necessary minimum standard of teleconsultation; for example. intravenous drug administration is only permitted if all required information is collected and all safety measures are taken (such as pulse oximetry, blood pressure measurement, ECG rhythm analysis). The tele-EMS physician evaluates the validity and reliability of the teleconsultation data transfer. In cases with low rates of data transfer and interruption of continuous patient monitoring, any delegation to the paramedics is not allowed according to the SOP if continuous monitoring is medically indicated. However, as is usual nationwide, the paramedics would continue to treat the patients, even in the case of complete interruption of the teleconsultation system. All paramedics are educated and trained in advanced life support. Furthermore, a backup mobile phone is available in each ambulance, in accordance with German industrial standard (DIN EN 1789) and the teleconsultation center is provided with a fixed-line connection in case of interruption of the Internet connection. An IT-safety concept was established in collaboration with the data protection officer of the city of Aachen to protect all transmitted and collected data against unauthorized access, unauthorized modifications and data loss. This IT-safety concept also comprises the storage time frame and the access rights.

AEs are possible regarding technical, organizational or medical factors. Occurrence of AEs during the teleconsultation must be documented on standardized forms provided to the tele-EMS physicians and the paramedics. These documentations must be analyzed immediately by the investigators, to recognize problems and potential hazards without delay and to initiate countermeasures as soon as possible. AEs listed under primary outcome measures are expected in connection with the specific kind of EMS care (conventional/telemedical).

### Definition of AEs

All unexpected AEs and the expected primary outcome AEs in the course of the EMS treatment must be documented on an AE Reporting Form which will be provided by the sponsor. All investigators will receive intensive training by the sponsor regarding the definition, documentation and reporting of AEs. Each AE must be specified as follows:

1. Duration
2. Severity (mild, moderate, or severe)
3. Causal relationship to the treatment (suspected/not suspected)
4. Required treatment of AE and action taken with trial drug
5. Outcome
6. Seriousness

### Definition of SAEs

A SAE is an AE which:

1. Results in death (i.e., is fatal)
2. Is immediately life-threatening
3. Results in persistent or significant disability/incapacity
4. Requires or prolongs patient hospitalization
5. Results in a congenital anomaly/birth defect
6. May jeopardize the patient and may require medical or surgical intervention to prevent one of the aforementioned outcomes (for example, intensive treatment in an emergency room without hospitalization)

SAEs must be reported by the principal investigator to the sponsor and the DSMC within 24 h after detection. All SAEs will be summarized in the sponsor’s Annual Safety Report.

### Ethics and dissemination

#### Research ethics approval

The study was presented to the Ethics Committee of the University of RWTH Aachen, 52074 Aachen, Germany. Approval with the reference number EK 170/15 was initially received on 23 November 2015. Protocol changes were approved on 20 September 2016. Any further changes to the study protocol, excluding changes for logistic and administration reasons or preventions of immediate hazards, must be approved by the Ethics Committee before their implementation.

### Protocol amendments

Any amendments, which may affect:

- Patient safety
- The integrity and credibility of data
- Changes in risk evaluation

Every substantial amendment will be submitted, after being signed by the principle investigator, the sponsor and the biostatistician, to the Ethics Committee for approval.

### Confidentiality

All included patients will be pseudonymized. A subject identification log will be managed according to the SOP of the sponsor and safely stored in the investigator’s site file. The investigators will collect all nonanonymized source data (e.g., the EMS Forms and the hospital database) and enter them pseudonymized manually in the e-CRF database. These data will only be handed over to a third party anonymized. The Department for Medical Statistics of the University Hospital RWTH Aachen will perform the statistical analysis of the anonymized data. All source data and documents will be stored for 10 years in locked cabinets in the Department of Anesthesiology of the University Hospital RWTH Aachen, with restricted access. Only the study team and, in connection with them, the monitors, auditors or the competent Ethics Committee will have access to personal data. In case of a patient withdrawing from study, only the anonymized data will be analyzed and stored. All regular (not study-specific) electronic patient data, which are used for documentation in the clinical routine, are stored on special safe servers and stored according to the legal requirements.

### Dissemination policy

The main results of this study will be published in a leading international journal. Further results of this study will be published in other professional English-language journals. The final report will be conducted in concordance with the Consolidated Standards of Reporting Trials (CONSORT) guideline as well as its extension to noninferiority trials. The study is already registered in the ClinicalTrials.gov registry (NCT02617875).

## Discussion

Increasing numbers of emergency calls, prolonged emergency response times by EMS physicians, and shortages of EMS physicians are having a big impact on the German EMS system. Furthermore, EMS treatment [33, 34] and documentation quality [35, 36] require urgent improvement.

There is good evidence for the better quality of supply with telemedicine networks between medical personnel and medical experts in prehospital emergency care as well as in other medical areas. The worldwide unique EMS teleconsultation system in Aachen was optimized and evaluated between 2009 and 2013 in nearly 1000 patient cases. The system appeared functional and was highly appreciated by the paramedics in Aachen [27]. In the majority of cases it reduced the requirement of EMS physicians and also enabled immediate medical advice to be given by EMS physicians to the paramedics. The “TemRas” project showed that an EMS teleconsultation system with a transregional teleconsultation center is feasible, can be operated without any complications, and that its requirements are in demand for daily use [7, 24]. Transfer of real-time patient vital data, pictures and audio communication system function between the paramedics on scene and the physician in the teleconsultation center was fast and reliable [26, 27, 37, 38]. This enabled prompt, safe and efficient patient treatment, even without an available physician on scene or until the arrival of the EMS physician. It enhanced the optimized use of the “resource” EMS physician. After its evaluation, it was implemented for routine use of the EMS system in Aachen. All conducted emergency missions with the aid of the tele-EMS physician were uneventful and provided a better quality of medical history, treatment and documentation. Furthermore, the physician engagement time was shortened by half. Until now, this system has only been used for the primarily dispatched ambulances staffed by paramedics, with restricted EMS-physician indications.

The need of EMS physicians for emergency missions has increased by about 2–3% per year over the last 20 years in Aachen. Before the implementation of the multifunctional mobile teleconsultation system an EMS physician was needed in 34–36% of emergency missions, now it has deceased to about 26% which is considerably less frequent than the national average [39].

There is a lack of evidence as to whether the advantages of the teleconsultation system, instead of a physically present physician on scene, could also be replicated in further cases with EMS-physician indications (excluding life-threatening emergency calls).

However, we would like to acknowledge some potential bias in our study: different levels of medical education or EMS experience within our physicians cannot be excluded during our study conducted in the clinical routine. In particular, our minimal educational requirement for the tele-EMS physician is beyond that for the conventional EMS physician according to the guideline of the General Medical Council of North Rhine-Westphalia. On the other hand, we also have conventional EMS physicians, with much more experience than our minimal educational requirement for the tele-EMS physician. This potential experience bias will be addressed in our statistical analysis with data presentation being at the medical education/experience level of our involved physicians in each group. Furthermore, the conventional EMS physician always has the opportunity to contact the tele-EMS physician for any advice which may be a further source of performance bias which will also be addressed in our data analysis.

### Risk-benefit assessment

So far, our experience with the teleconsultation EMS system shows at least an equivalent diagnostic and treatment quality between the tele-EMS physician and the conventional physically present EMS physician on scene. As immediate life-threatening emergency cases (as described in the “Exclusion criteria”) are excluded from this study, no specific risk is expected with either with conventional treatment or with telemedical treatment.

### Trial status

Patient recruitment is expected to start in March 2017. The predicted study recruiting end-date is March 2018.

## Additional files

Additional file 1: SPIRIT Checklist. (DOC 123 kb)
Additional file 2: Participant timeline: Standard Protocol Items: Recommendations for Interventional Trials (SPIRIT) diagram. (PDF 123 kb)

## Abbreviations

AE: Adverse event
AHA: American Heart Association
CEC: Clinical Endpoint Committee
COPD: Chronic obstructive pulmonary disease
CRF: Case Report Form
CTC-A: Clinical Trial Center Aachen
DFG: German Research Foundation
DIN: German industrial standard
DIVI: German Interdisciplinary Society for Intensive and Emergency Medicine
DSMC: Data Safety Monitoring Committee
ECG: Electrocardiogram
e-CRF: Electronic Case Report Form
EMS: Emergency Medical Service
ICD 10: *International Classification of Diseases 10*
ICH-GCP: International Conference on Harmonization of Technical Requirements for Registration of Pharmaceuticals for Human Use-Good Clinical Practice
ICU: Intensive Care Unit
IT: Information technology
RettG NRW: Law on Rescue Services of the state of North Rhine-Westphalia
SAE: Serious adverse event
SOP: Standard operating procedures
STEMI: ST-elevation myocardial infarction
StGB: German Penal Code
tele-EMS physician: Teleconsultation EMS physician

## References

[1] Roessler M, Zuzan O (2006). EMS systems in Germany. Resuscitation.

[2] Leistungen des Rettungsdienstes 2012/2013. http://www.bast.de/DE/Publikationen/Foko/Downloads/2015-15.pdf?__blob=publicationFile&v=3. Accessed 20 Mar 2016.

[3] Reimann B, Maier BC, Lott R, Konrad F (2004). Gefährdung der Notarztversorgung im ländlichen Gebiet. Notfall Rettungsmedizin.

[4] Behrendt H, Schmiedel R, Auerbach K (2009). Überblick über die Leistungen des Rettungsdienstes in der Bundesrepublik Deutschland im Zeitraum 2004/05. Notfall + Rettungsmedizin.

[5] Bericht über Maßnahmen auf dem Gebiet der Unfallverhütung im Straßenverkehr 2012 und 2013. http://dip21.bundestag.de/dip21/btd/18/024/1802420.pdf. Accessed 22 Feb 2016.

[6] Schmiedel R, Behrendt H. Leistungen des Rettungsdienstes 2008/09. Analyse des Leistungsniveaus im Rettungsdienst für die Jahre 2008 und 2009. http://www.bast.de/DE/Publikationen/Berichte/unterreihe-m/2011-2010/m217.html. Accessed 20 Feb 2016.

[7] Luiz T, van Lengen RH, Wickenkamp A, Kranz T, Madler C (2011). Operational availability of ground-based emergency medical services in Rheinland-Palatinate: state-wide web-based system for collation, display and analysis. Anaesthesist.

[8] Notfall: Notkompetenz II. http://www.bundesaerztekammer.de/richtlinien/empfehlungenstellungnahmen/notfall-notkompetenz-ii/. Accessed 23 Mar 2016.

[9] Audebert HJ, Kukla C, Clarmann von Claranau S, Kühn J, Vatankhah B, Schenkel J (2005). Telemedicine for safe and extended use of thrombolysis in stroke: the Telemedic Pilot Project for Integrative Stroke Care (TEMPiS) in Bavaria. Stroke.

[10] Audebert HJ, Schenkel J, Heuschmann PU, Bogdahn U, Haberl RL, Telemedic Pilot Project for Integrative Stroke Care Group (2006). Effects of the implementation of a telemedical stroke network: the Telemedic Pilot Project for Integrative Stroke Care (TEMPiS) in Bavaria, Germany. Lancet Neurol.

[11] Audebert HJ, Schultes K, Tietz V, Heuschmann PU, Bogdahn U, Haberl RL (2009). Long-term effects of specialized stroke care with telemedicine support in community hospitals on behalf of the Telemedical Project for Integrative Stroke Care (TEMPiS). Stroke.

[12] Demaerschalk BM, Bobrow BJ, Raman R, Kiernan T-EJ, Aguilar MI, Ingall TJ (2010). Stroke team remote evaluation using a digital observation camera in Arizona: the initial Mayo Clinic Experience trial. Stroke.

[13] Meyer BC, Raman R, Hemmen T, Obler R, Zivin JA, Rao R (2008). Efficacy of site-independent telemedicine in the STRokE DOC trial: a randomised, blinded, prospective study. Lancet Neurol.

[14] Dhruva VN, Abdelhadi SI, Anis A, Gluckman W, Hom D, Dougan W (2007). ST-Segment Analysis Using Wireless Technology in Acute Myocardial Infarction (STAT-MI) trial. J Am Coll Cardiol.

[15] Adams GL, Campbell PT, Adams JM, Strauss DG, Wall K, Patterson J (2006). Effectiveness of prehospital wireless transmission of electrocardiograms to a cardiologist via hand-held device for patients with acute myocardial infarction (from the Timely Intervention in Myocardial Emergency, NorthEast Experience [TIME-NE]). Am J Cardiol.

[16] Sanchez-Ross M, Oghlakian G, Maher J, Patel B, Mazza V, Hom D (2011). The STAT-MI (ST-Segment Analysis Using Wireless Technology in Acute Myocardial Infarction) trial improves outcomes. JACC Cardiovasc Interv.

[17] Terkelsen CJ, Nørgaard BL, Lassen JF, Gerdes JC, Ankersen JP, Rømer F (2002). Telemedicine used for remote prehospital diagnosing in patients suspected of acute myocardial infarction. J Intern Med.

[18] Sejersten M, Sillesen M, Hansen PR, Nielsen SL, Nielsen H, Trautner S (2008). Effect on treatment delay of prehospital teletransmission of 12-lead electrocardiogram to a cardiologist for immediate triage and direct referral of patients with ST-segment elevation acute myocardial infarction to primary percutaneous coronary intervention. Am J Cardiol.

[19] Skorning M, Bergrath S, Rörtgen D, Brokmann JC, Beckers SK, Protogerakis M (2009). E-health in emergency medicine—the research project Med-on-@ix. Anaesthesist.

[20] Ziegler V, Rashid A, Müller-Gorchs M, Kippnich U, Hiermann E, Kögerl C (2008). Mobile computing systems in preclinical care of stroke. Results of the Stroke Angel initiative within the BMBF project PerCoMed. Anaesthesist.

[21] Ting HH, Krumholz HM, Bradley EH, Cone DC, Curtis JP, Drew BJ (2008). Implementation and integration of prehospital ECGs into systems of care for acute coronary syndrome: a scientific statement from the American Heart Association Interdisciplinary Council on Quality of Care and Outcomes Research, Emergency Cardiovascular Care Committee, Council on Cardiovascular Nursing, and Council on Clinical Cardiology. Circulation.

[22] Schwamm LH, Audebert HJ, Amarenco P, Chumbler NR, Frankel MR, George MG (2009). Recommendations for the implementation of telemedicine within stroke systems of care: a policy statement from the American Heart Association. Stroke.

[23] Schwamm LH, Holloway RG, Amarenco P, Audebert HJ, Bakas T, Chumbler NR (2009). A review of the evidence for the use of telemedicine within stroke systems of care: a scientific statement from the American Heart Association/American Stroke Association. Stroke.

[24] Brokmann JC, Rossaint R, Bergrath S, Valentin B, Beckers SK, Hirsch F (2015). Potential and effectiveness of a telemedical rescue assistance system. Prospective observational study on implementation in emergency medicine. Anaesthesist.

[25] Greb I, Wranze E, Hartmann H, Wulf H, Kill C (2011). Analgesie beim Extremitätentrauma durch Rettungsfachpersonal. Notfall + Rettungsmedizin.

[26] Bergrath S, Czaplik M, Rossaint R, Hirsch F, Beckers SK, Valentin B (2013). Implementation phase of a multicentre prehospital telemedicine system to support paramedics: feasibility and possible limitations. Scand J Trauma Resusc Emerg Med.

[27] Czaplik M, Bergrath S, Rossaint R, Thelen S, Brodziak T, Valentin B (2014). Employment of telemedicine in emergency medicine. Clinical requirement analysis, system development and first test results. Methods Inf Med.

[28] O’Brien PC, Fleming TR (1979). A multiple testing procedure for clinical trials. Biometrics.

[29] Matilde Sanchez M, Chen X (2006). Choosing the analysis population in non-inferiority studies: per protocol or intent-to-treat. Stat Med.

[30] Haynes AB, Weiser TG, Berry WR, Lipsitz SR, Breizat A-HS, Dellinger EP (2011). Changes in safety attitude and relationship to decreased postoperative morbidity and mortality following implementation of a checklist-based surgical safety intervention. BMJ Qual Saf.

[31] Haynes AB, Weiser TG, Berry WR, Lipsitz SR, Breizat A-HS, Dellinger EP (2009). A surgical safety checklist to reduce morbidity and mortality in a global population. N Engl J Med.

[32] Weiser TG, Haynes AB, Dziekan G, Berry WR, Lipsitz SR, Gawande AA (2010). Effect of a 19-item surgical safety checklist during urgent operations in a global patient population. Ann Surg.

[33] Timmermann A, Russo SG, Eich C, Roessler M, Braun U, Rosenblatt WH (2007). The out-of-hospital esophageal and endobronchial intubations performed by emergency physicians. Anesth Analg.

[34] Qualitätssicherung im Rettungsdienst Baden-Württemberg—Downloads. http://www.sqrbw.de/90.php. Accessed 2 May 2016.

[35] Bergrath S, Rörtgen D, Skorning M, Fischermann H, Beckers SK, Mutscher C (2011). Emergency mission documentation in simulated care. Video-based error analysis. Anaesthesist.

[36] Bergrath S, Skorning M, Rörtgen D, Beckers SK, Brokmann JC, Mutscher C (2011). Is paper-based documentation in an emergency medical service adequate for retrospective scientific analysis? An evaluation of a physician-run service. Emerg Med J.

[37] Bergrath S, Reich A, Rossaint R, Rörtgen D, Gerber J, Fischermann H (2012). Feasibility of prehospital teleconsultation in acute stroke—a pilot study in clinical routine. PLoS One.

[38] Bergrath S, Rörtgen D, Rossaint R, Beckers SK, Fischermann H, Brokmann JC (2011). Technical and organisational feasibility of a multifunctional telemedicine system in an emergency medical service—an observational study. J Telemed Telecare.

[39] Rettungsdienstbedarfsplan 2014 der Stadt Aachen. www.aachen.de-Rettungsdienstbedarfsplan, http://www.aachen.de/DE/stadt_buerger/politik_verwaltung/feuerwehr/downloads/rettungsdienst/rettungsdienst_2014/index.html. Accessed 20 May 2016.

[40] Farrington CP, Manning G (1990). Test statistics and sample size formulae for comparative binomial trials with null hypothesis of non-zero risk difference or non-unity relative risk. Stat Med.

