# Supplementary material for: Telemedical support for prehospital Emergency Medical Service (TEMS trial): study protocol for a randomized controlled trial
Source: Trials. 2017 Jan 26;18:43. doi: 10.1186/s13063-017-1781-2 (PMC5270339; doi:10.1186/s13063-017-1781-2)
Supplement: Additional file 2: — Participant timeline: Standard Protocol Items: Recommendations for Interventional Trials (SPIRIT) diagram. (PDF 123 kb) [file 13063_2017_1781_MOESM2_ESM.pdf]

		Main STUDY PERIOD							
		Enrolment	Allocation	Post-allocation				Close-out	
**PHASE***	**Run-In phase**	***A***	***B***	***C***	***D***		***E***		***F***
**TIMEPOINT****	$-t_2$	$-t_1$	**0**	t_1	t_2	t_3	t_4	t_5	t_x
**ENROLMENT:**									
**Eligibility screen**	X	X							
**Informed consent**	X			◆——	——	——◆			
**Allocation**	X		X						
**INTERVENTIONS:**									
Conventional EMS physician treatment	X			X					
Tele-EMS physician treatment	X			X					
**ASSESSMENTS:**									
a) Screening for patient related exclusion criteria:<br>*• Apnea*<br>*• Acute respiratory failure*<br>*• Cardio-circulatory arrest*<br>*• ST-elevation myocardial infarction (STEMI)*<br>*• Unconsciousness*<br>*• Persistent seizure*<br>*• Life- threatening rhythm disorder*<br>*• Major trauma*<br>*• Complex psychiatric disorders*<br>*• Age < 18 years*<br><br>***b) Screening for emergency case related exclusion criteria:***<br>*• Major vehicle accident*<br>*• (Traffic) accident with children*<br>*• Fall from a height (> 3m)*<br>*• Gunshot-, stab-, or blow injuries in the head, neck and torso area*<br>*• Fires with reference to*	X	X							

personal injury<br>*• Explosion-, thermic or chemical accidents with reference to personal severe injury*<br>*• High-voltage electrical accident*<br>*• Water connected accidents (drowning-, diving accident, fall through ice)*<br>*• Accidents involving hazardous goods*<br>*• Hostage-taking, rampage or other crimes with potential danger for human life (preventive deployment, police consultation)*<br>*• Immediate threatening suicide*<br>*• Immediate forthcoming delivery or preceding delivery*									
Primary outcome variables<br><br>*• Immediate allergic reaction*<br>*• Intervention-related and immediate treatment-requiring blood pressure drop on scene*<br>*• Immediate intervention-related apnea or respiratory insufficiency on scene*	X			X					
• Intervention-related circulatory arrest within 24h of EMS treatment	X			X	X				
Secondary outcome variables<br><br>***•*** *Quality of medical history survey*<br>*• Treatment quality*<br>*• Quality of documentation*<br>*• Duration of the physician engagement-time.*<br>*• Fulfillment of predefined quality indicators for "Tracer" diagnoses (trauma, stroke, acute coronary syndrome, pain*	X			X					

control, bronchial asthma, chronic obstructive pulmonary disease, seizure, sepsis, hypoglycemia)<br>*• Correct pre-hospital diagnosis*<br>*• Other adverse events*<br>*• Premature termination of the telemedical or conventional EMS operation, as unnecessary*<br>*• Required conversion from the primary dispatched tele-EMS physician to a conventional EMS physician*<br>*• Assessment if a conventional EMS physician operation could have been handled by a tele-EMS physician*									
Other Outcome Measures<br><br>*• Time point of the first contact with a physician, time span between the emergency call and hospital arrival*<br>*• Seven-step National Advisory Committee for Aeronautics severity score*<br>*• Proportion of conventional emergency cases, which were passed to a tele-EMS physician*<br>*• Necessity of an EMS physician at all*<br>*• Technical performance*<br>*• Frequency of tele-EMS contacting by a conventional EMS physician*<br>*• Level of medical education/ experience of the involved physicians in each group*	X			X					
• Survey of the patients satisfaction with the	X			◆	—	◆			

used EMS system										
• *Death within 24 hours and until day 30 of hospitalization respectively until discharge from hospital*	X			◆	—	—	◆			
• *Discharge destination from hospital* • *Intensive Care Unit (ICU) and hospital length of stay*	X					X				
• *Death within 30 and 90 days after EMS treatment*	X						X		X	
• *Retrospective analysis of the same* t_1- t_3 *data for the excluded (non-randomized) conventional EMS physician cases (collected from the conventional EMS physician protocols and the hospital database)*										X
Survey of the paramedics, the EMS physicians and the emergency room personnel in regard to their satisfaction with the tele-EMS system (once/ twice during the study period)										X

* Specific phases: Run-In phase (1.5-2 months prior study start), A = Enrolment, B = Allocation, C = During EMS intervention, D = Early follow up, E = Late follow up, F = Additional analysis
Specific timepoints: $-t_2$ = Run-In phase (1.5-2 months prior study start), $-t_1$ = Enrolment during the emergency call in the dispatching center, ***0* = Allocation via automatic randomization by the dispatching software, t_1 = EMS operation on scene, t_2 = 24 hours after EMS intervention, t_3 = Discharge from hospital, t_4 = Follow-up 30 days after EMS intervention, t_5 = Follow up 90 days after EMS intervention, t_x = Parallel to the main study.
